# Therapeutic Liquid Formulations Based on Low Transition Temperature Mixtures for the Incorporation of Anti-Inflammatory Drugs

**DOI:** 10.3390/pharmaceutics13101620

**Published:** 2021-10-05

**Authors:** Ana Roda, Alexandre Paiva, Ana Rita C. Duarte

**Affiliations:** LAQV, REQUIMTE, Chemistry Department, NOVA School of Science and Technology, 2829-516 Caparica, Portugal; a.roda@fct.unl.pt (A.R.); alexandre.paiva@fct.unl.pt (A.P.)

**Keywords:** LTTM, NSAID, therapeutic liquid formulations, solubility, celecoxib

## Abstract

Most nonsteroidal anti-inflammatory drugs (NSAIDs) present poor aqueous solubility, impairing their efficiency in physiological media. In this context, Low Transition Temperature Mixtures (LTTMs) are a promising platform to overcome drugs’ poor solubility, forming therapeutic liquid formulations. In this work, the LTTMs of citric acid:L-arginine:water (C:A:W) and glycerol:sorbitol (G:S) were studied in terms of their features and assessed in terms of their ability to increase the solubility of six NSAIDs in physiological media. The physicochemical properties of LTTMs were characterized by state-of-art techniques commonly used for these systems. The cytotoxicity of G:S was also evaluated in L929 mouse fibroblasts and the viscosity, polarity, and pH properties of the studied mixtures were related to the solubility of NSAIDs. The pH and polarity were the parameters that most influenced the drugs’ solubility. Ibuprofen, naproxen, ketoprofen, indomethacin, and flurbiprofen did not present any solubility improvement in the formulations tested. However, concentrated mixtures of C:A:W or G:S in the physiologic-mimicked media (PBS) rendered a celecoxib solubility 4 and 5 times higher than PBS, respectively. These therapeutic liquid formulations of celecoxib in C:A:W or G:S can be a promising tool to increase celecoxib’s therapeutic efficiency in local applications.

## 1. Introduction

Nonsteroidal anti-inflammatory drugs (NSAIDs) are anti-inflammatory, antipyretic, and analgesic medicines commonly used worldwide for the symptomatic relief of headaches, strains, sprains, viral infections such as the flu or corona, arthritis, and other cases of pain, fever, and/or inflammation [1]. Despite being regarded as generally safe, most NSAIDs require the intake of higher amounts than the therapeutic dose due to their general low solubility in the physiological media [2]. Further, these drugs are associated to gastrointestinal, cardiovascular, and other adverse effects potentiated by high doses and prolonged consumption [1]. In the case of chronic diseases like osteoarthritis, this is specially concerning as this is the most administered treatment for the persistent symptoms of pain and inflammation [3,4,5]. In this context, biocompatible formulations that improve the efficiency and safety of marketed drugs, while avoiding the long-term development of new drugs associated to the regulatory entities, are of great importance.

Recently, the platform of LTTMs, including deep eutectic systems (DES) and therapeutic DES (THEDES), has been presented as a promising alternative strategy for drugs’ delivery by increasing their solubility [6,7,8,9,10,11,12,13] and/or their biological activity [13,14,15]. As expressed by the name, LTTMs present low-temperature transitions, which are commonly liquid at room temperature [16]. Their liquefaction is solely promoted by interactions between their components, not involving any chemical reaction and rendering 100% atom economy, which complies with several green metrics. Moreover, their constituents can be chosen from non-toxic, biodegradable, and biocompatible molecules, as required for pharmaceutical products [17]. Envisaging therapeutic applications, drugs can be incorporated in LTTMs, either as a starting material or by solubilization. These types of therapeutic formulations have been described as propitious to improve drugs’ bioavailability by facilitating their permeation, dissolution, and absorption [7,8,9,10,11,12,18,19,20]. Formulations with increased drug solubility post-administration are highly promising as drug dosage can be minimized up to the effective amount, reaching therapeutic efficiency with lower drug intakes and reduced adverse effects.

Regarding NSAIDs, some studies already reported their improved solubility in LTTMs comparatively to aqueous media and other conventional solvents. Lu and co-authors reported 17 salt-based LTTM formulations in which the solubility of several NSAIDs was increased 5- to 17-fold in respect to their solubility in aqueous media [21]. In a different study, Hyun et al. studied eutectic mixtures of celecoxib, a NSAID, with adipic acid or saccharin. In comparison to aqueous media or the respective physical mixtures, the eutectics significantly improved the dissolution rate of celecoxib and its wettability [22]. Furthermore, Mokhtarpour and colleagues published a study involving four NSAIDs and two other drugs, with a solubility enhancement of 127- to 136,444-fold when dissolved in five choline chloride-based LTTMs instead of water. Their experimental results were in accordance with the empiric solubility predictions performed using Hansen solubility parameters (HSP) [23]. An additional work, by Palmelund and co-workers, studied the solubility of 11 drugs, including five NSAIDs, in six LTTMs, four of which were choline chloride-based and two were of betaine:glycerol:water and lactic acid:glucose:water. The drugs’ solubility in these systems was compared with four conventional solvents, water, ethanol, glycerol, and polyethylene glycol 300 (PEG300). In most cases, the LTTMs enhanced the drugs’ solubility in respect to water but not in comparison to the other conventional solvents. Moreover, using the computational Conductor-like Screening Model for Real Solvents (COSMO-RS), the authors achieved good predictions of the experimental solubilities [24]. The empiric and computational solubility predictors used by Mokhtarpour et al. [23] and Palmelund et al. [24], respectively, may be powerful tools to screen several LTTM systems, in regards to their solvation potential for certain drugs. Still, to the best of our knowledge, few studies on LTTMs and NSAIDs have yet been published, with the ones herein summarized being representative of the progress on the topic. Despite the positive outputs of LTTMs improving NSAIDs’ solubility, few therapeutic combinations were studied, most of which were based on choline chloride, which may be toxic [25,26], as well as the betaine:glycerol [27]. Therefore, different and diverse therapeutic formulations must be designed, studied, and tailored for the required application(s).

In this work, LTTMs of glycerol:sorbitol (G:S) and citric acid:L-arginine:H_2_O (C:A:W) were explored, namely, in terms of their properties and influence in the solubilization of NSAIDs in physiological media. Concentrated formulations of G:S and C:A:W showed potential to increase the solubility of some NSAIDs. The therapeutic system based on G:S was particularly interesting for local administrations with sustained drug delivery.

## 2. Materials and Methods

### 2.1. Materials

Citric acid monohydrate ≥99.5% [5949-29-1] was purchased from PanReac AppliChem (Barcelona, Spain) and glycerol 99.5% [58-81-5] from Scharlau (Barcelona, Spain). Sorbitol ≥98% [50-70-4], L-arginine ≥98% [74-79-3], Reichardt’s dye 90% [10081-39-7], Nile red ≥98% [7385-67-3], and phosphate buffer saline (PBS) tables were acquired from Sigma Aldrich (St. Louis, MO, USA). PBS solution was prepared in deionized water (0.01 M phosphate buffer, 0.0027 M potassium chloride, 0.137 M sodium chloride, pH 7.4, 25 °C) and stored at 4 °C. Dimethyl sulfoxide-d6 (DMSO-d6, 99.96% D) [2206-27-1] was bought from Eurisotop (Essonne, France). The mouse fibroblast L929 cell line was provided by the DSMZ Catalogue of Human and Animal Cell Lines (ACC2, Braunschweig, Germany) [28]. For the cell media, Minimum Essential Medium (MEM, with 1.5 g/L sodium bicarbonate, non-essential aminoacids, L-glutamine, and sodium pyruvate), Fetal Bovine Serum (FBS), trypsin 0.25%, and antibiotic-antimycotic solution (10,000 U/mL penicillin, 10 mg/mL streptomycin, and 0.025 mg/mL amphotericin) were obtained from Corning (Corning, NY, USA). The 3-(4,5-dimethylthiazol-2-yl)-5-(3-carboxymethoxyphenyl)-2-(4-sulfophenyl)-2H-tetrazolium (MTS, <2%) solution was purchased from Promega (Madison, WI, USA). The NSAIDs celecoxib ≥98% [169590-42-5], naproxen [22204-53-1], ketoprofen ≥98% [22071-15-4], and flurbiprofen ≥98.5% [5104-49-4] were purchased from Sigma Aldrich (St. Louis, MO, USA), whereas Ibuprofen 99% [15687-27-1] was bought from AlfaAesar (Haverhill, MA, USA) and Indomethacin >98% [53-86-1] from TCI Chemicals (Tokyo, Japan). The acetonitrile [75-05-8], phosphoric acid [7664-38-2], and potassium dihydrogen phosphate [7778-77-0] used for high-performance liquid chromatography (HPLC) were provided by Carlo Erba (Milano, Italy), Fluka (Buchs, Switzerland) and Sigma-Aldrich (St. Louis, MO, USA), respectively. The buffers of phosphoric acid 0.2% (50:50, *v*/*v*) and potassium dihydrogen phosphate 50 mM 50:50 (*v*/*v*), pH 4.2 were prepared in deionized water.

### 2.2. Preparation of Liquid Formulations

Liquid systems with a molar ratio of 2:1 glycerol:sorbitol (G:S) and 1:1:7 citric acid:L-arginine:H_2_O (C:A:W) [29] were prepared. Briefly, the components were mixed in their respective ratio and submitted to heating and stirring until a translucid liquid was obtained. The water weight was controlled and confirmed to be constant for both mixtures (G:S = 0.15 ± 0.06%, Karl fisher; C:A:W = 26.24 ± 0.51%).

### 2.3. Differential Scanning Calorimetry (DSC)

Thermal events were analysed using a DSC Q200 from TA Instruments Inc. (New Castle, DE, USA).The samples were sealed in aluminium hermetic pans/lids and equilibrated at 40 °C. The samples were then cooled up to −90 °C, maintained for 5 min, and heated up to 150 °C. A constant heating/cooling rate of 10 °C/min and a nitrogen flow rate of 50 mL were applied. The thermal events were determined using the TA Universal Analysis 2000 (4.7.0.2) software.

### 2.4. Nuclear Magnetic Resonance

Proton nuclear magnetic resonance (^1^H NMR) spectra were recorded on a Bruker Avance III 400 spectrometer (Bruka, Billerica, MA, USA) at 400 MHz. Spectra were obtained from about 400 µL of each sample with 50 µL of DMSO-d6. Chemical shifts were expressed in ppm. MestReNova software (11.0.4-18998) was used for data analysis.

### 2.5. Attenuated Total Reflection-Fourier Transform Infrared (ATR-FTIR)

ATR-FTIR was performed on a PerkinElmer Spectrum Two (Waltham, MA, USA) with a universal ATR accessory with diamond crystals. Spectra with 4 cm^−1^ resolution were acquired at room temperature from 16 scans in Transmittance mode (% T) and in a wavenumber range of 400–4000 cm^−1^.

### 2.6. Cytotoxicity

The cytotoxicity was performed in a mouse fibroblast L929 cell line, according to the ISO/EN 10993 guideline [30]. Cells were cultured in MEM media supplemented with 10% FBS and 1% of antibiotic-antimycotic solution. Cultures were maintained in a humidified incubator with 5% of CO_2_, at 37 °C. Cell viability was determined through MTS colorimetric assay, for which non-supplemented MEM was used. Cell monolayers with an initial concentration of 1 × 10^4^ cells/100 µL were seeded in a 96-well plate for 24 h. Several concentrations of the samples were prepared in non-supplemented MEM from stock solutions of 0.84, 0.6 and 0.278 g/mL for glycerol, glycerol:sorbitol mixtures and sorbitol, respectively. The cell media was then removed and 100 µL of the different sample concentrations were added. After an incubation period of 24 h, samples were removed and the cells washed with PBS. The MTS reagent, previously diluted 62.5-fold in non-supplemented MEM media, was then added. Upon 3 h of incubation, the absorbance (Abs) was measured at 490 nm in a VICTOR Nivo™ microplate reader (PerkinElmer, Waltham, MA, USA). Three independent experiments were performed for each sample, analysed in triplicate. The percentage of cell viability was calculated as follows:

(1) $$\mathrm{Viability}\;(\%)\;=\frac{\;\mathrm{Abs}\;(\mathrm{sample})}{\mathrm{Abs}\;(\mathrm{control})}\;\times 100$$

The 50% inhibitory concentration (IC_50_) was interpolated from the non-linear regression of the log (concentration) versus viability using GraphPad Prism 8.0.1 software and expressed as mean ± SD.

### 2.7. Viscosity

The viscosity of the liquid systems was measured using a rheometer (Anton Paar MCR102, Graz, Austria) with a 50-mm stainless-steel parallel plate geometry. The samples were loaded to the plate, equilibrated at the initial analysis temperature, and analysed at a constant shear stress of 20 Pa, within a temperature range between 4 and 80 °C at a rate of 10 °C/min. Measurements were made in triplicate and the results expressed as mean of dynamic viscosity ± SD.

### 2.8. Polarity

Polarity analysis was performed using Nile red and Reichardt’s dye as solvatochromic probes. Stock solutions of 1 mg/mL and 5 mg/mL were prepared in methanol for Nile red and Reichardt’s dyes, respectively. Ten µL of Nile red or 100 µL of Reichardt’s dyes were added to about 1 g of C:A:W, G:S, or H_2_O. The polarity of H_2_O was measured for comparison, instead of PBS, as the salts of PBS interfere with the dyes used. Their maximum wavelength ($\lambda_{\max})\;$ was obtained from UV spectra and used to calculate their energy transition (E_T_) values and establish their relative polarities, according to [31,32]. E_T_ values were expressed as the mean ± SD of three replicates.

(2) $$E_{T}=\frac{\;28591}{\lambda_{\max}}\;\mathrm{kcal}/\mathrm{mol}\;$$

### 2.9. pH

The pH measurements of the LTTMs in PBS were carried out at room temperature using an analogic pH meter (914 pH/Conductometer, 2.914.0220, Metrohm, Herisau, Switzerland) with a glass electrode coupled to a temperature sensor (NTC, 6.0228.010, Metrohm). The results presented are the average of triplicates and represented as mean ± SD.

### 2.10. NSAIDs’ Solubility

NSAIDs were added in excess to G:S and C:A:W and stirred at 100 rpm and 37 °C for 24 h. Different volumes of fresh PBS were then added to the G:S and C:A:W to achieve a final ratio of LTTM:PBS (*w*/*w*) of 2:1, 1:1, or 1:10. These mixtures were stirred at the same conditions for another 24 h. The solubility of the NSAIDs in each mixture was determined by high-performance liquid chromatography (HPLC). For this, the mixtures were filtered (0.22 µm PTFE hydrophilic) and stored at 4 °C until further analysis by HPLC. The solubility of the NSAIDs in PBS (physiologic mimic) was also tested as control. PBS was saturated with each NSAID and stirred for 48 h at 37 °C, 100 rpm. Solubility was then determined by HPLC, following the same procedure as before. Each solubility assay was performed at least in triplicate.

The NSAIDs’ solubility was quantified by HPLC, according to methods adapted for indomethacin [33] and for ibuprofen, naproxen, ketoprofen, flurbiprofen, and celecoxib [34]. Briefly, 10 µL of indomethacin samples were analysed in an Agilent 1100 Series HPLC (Agilent, Santa Clara, CA, USA) using a ZORBAX SB-Phenyl column (250 × 4.6 mm 5 µm, Agilent, Santa Clara, CA, USA) with isocratic elution at a flow of 1 mL/min in acetonitrile:phosphoric acid 0.2% (50:50, *v*/*v*) and detection at 237 nm in a UV-vis detector (Agilent, Santa Clara, CA, USA). In turn, 20 µL of celecoxib, naproxen, ketoprofen, flurbiprofen, or ibuprofen samples were analysed in a Smartline HPLC Series (Knauer, Berlin, Germany), eluted in a Keystone Kromasil C18 column (250 × 4.6 mm 5 µm, Thermo Scientific, Waltham, MA, USA) with acetonitrile:potassium dihydrogen phosphate 50 mM 50:50 (*v*/*v*), pH 4.2, at an isocratic flow of 0.6 mL/min, and detected at 220 nm in a Smartline 2500 UV-detector (Knauer, Berlin, Germany). All analyses were performed at 25 °C.

The calibration curves were prepared by dilution of the drug in eluent at the following linear ranges: indomethacin 5.2–260 mg/L; celecoxib 0.14–142 mg/L; naproxen 0.31–31 mg/L; ketoprofen 0.12–58.5 mg/L; flurbiprofen: 11.4–114 mg/L; ibuprofen 12–60 mg/L. A known concentration of drug in PBS was tested as control to assure the method feasibility. The G:S and C:A:W mixtures were also tested in the methods used. No interference of these mixtures was observed.

### 2.11. Statistical Analysis

Statistical analysis was performed using GraphPad Prism 8.0.1 software. Normal distribution was evaluated by Shapiro–Wilk test. The viability profiles of G:S and the respective physical mixture (G + S) did not follow a normal distribution. Thus, they were compared through the non-parametric Mann–Whitney test. Regarding the solubility data, they followed a normal distribution, being submitted to one-way NOVA analysis. Statistically significant differences were considered for *p* ≤ 0.05.

## 3. Results and Discussion

### 3.1. Preparation of LTTMs

The G:S and C:A:W systems were chosen based on their favourable properties towards osteoarthritis (OA) treatment, which was chosen as a model application. In the case of C:A:W, it contains L-arginine that has been reported as anti-inflammatory adjuvant in human osteoarthritic cells [35]. Moreover, it was also reported to increase the synthesis and deposition of collagen [36], an important component of cartilage whose wear is the main cause of OA symptoms. A molar ratio of C:A:W 1:1:7 was chosen based on the results from previous studies with this mixture [18].

Regarding G:S, to the best of our knowledge, this is the first study reporting this mixture. Four molar ratios were tested (1:2, 1:1, 2:1, and 3:1) but only the 2:1 and 3:1 formed translucid liquids at room temperature. The G:S 2:1 molar ratio was chosen as it was observed to gelify over time (when resting at room temperature in a glass vial). Even though this effect was not explored under the scope of this work, it may be of interest and suitable for the development of local, sustained delivery formulations. To guarantee consistent initial properties of G:S 2:1, these samples were equilibrated prior to use at 80 °C.

Given that only one molar ratio of each system (C:A:W 1:1:7 and G:S 2:1) was herein studied, these mixtures were identified as C:A:W and G:S throughout the text.

The properties of the selected LTTMs are presented in the following sections. Their physicochemical properties were first studied by DSC, NMR, and FTIR and the biological properties, namely, the cytotoxicity, was evaluated. Other properties such as viscosity, polarity, and pH were explored and correlated with the NSAIDs’ solubility to provide insights on the mechanisms that influence drugs’ solubility.

### 3.2. Thermal Characterization

DSC is often used in the characterization of LTTMs, as they are commonly defined as mixtures with low-temperature transitions, either glass transition temperatures (T_g_) and/or melting temperatures (T_m_). The DSC results for the C:A:W system were previously reported by us (see Figure S1 in supplementary material) [29]. From those results, although a melting peak around −13 °C was detected for this system, it was attributed to the free water component of the mixture. Thus, in the conditions tested, no T_m_ corresponding to the ternary mixture was observed.

**Figure 1:**
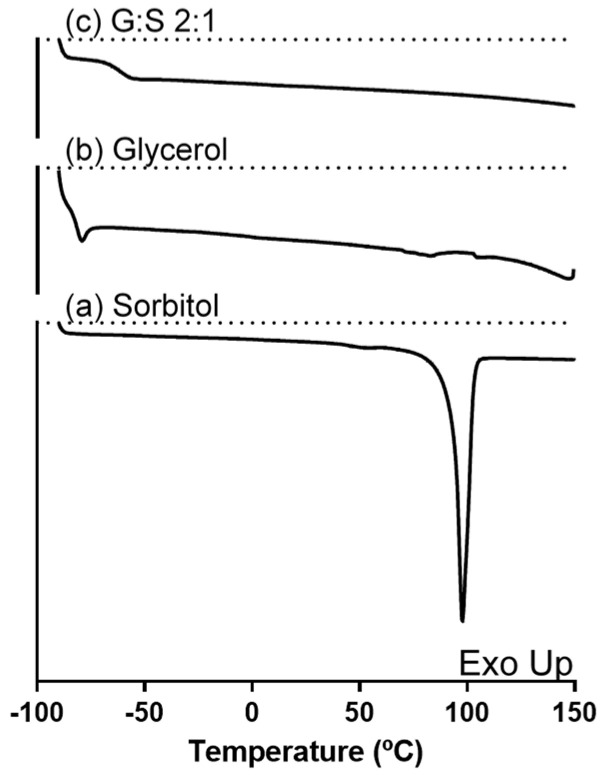
DSC thermogram of (**a**) sorbitol, (**b**) glycerol, and (**c**) G:S 2:1 molar ratio.

Regarding the system G:S and its pure components, the thermal events are represented in Figure 1. As observed, pure sorbitol showed a T_m_ around 98 °C, in accordance with other values reported in literature for its stable form [37,38,39]. In turn, no melting events were possible to acquire for pure glycerol or the G:S system, only their T_g_ of −81 °C and −62 °C, respectively, were detected. The T_g_ measured for glycerol was concordant with Zondervan and co-workers [40]. Although glycerol has a T_m_ value around 17 ± 4 °C [41], it has been described to behave as a supercooled liquid due to its high viscosity [40], which is in accordance with the lack crystallization and subsequent melting herein observed.

### 3.3. Intermolecular Interactions

**Figure 2:**
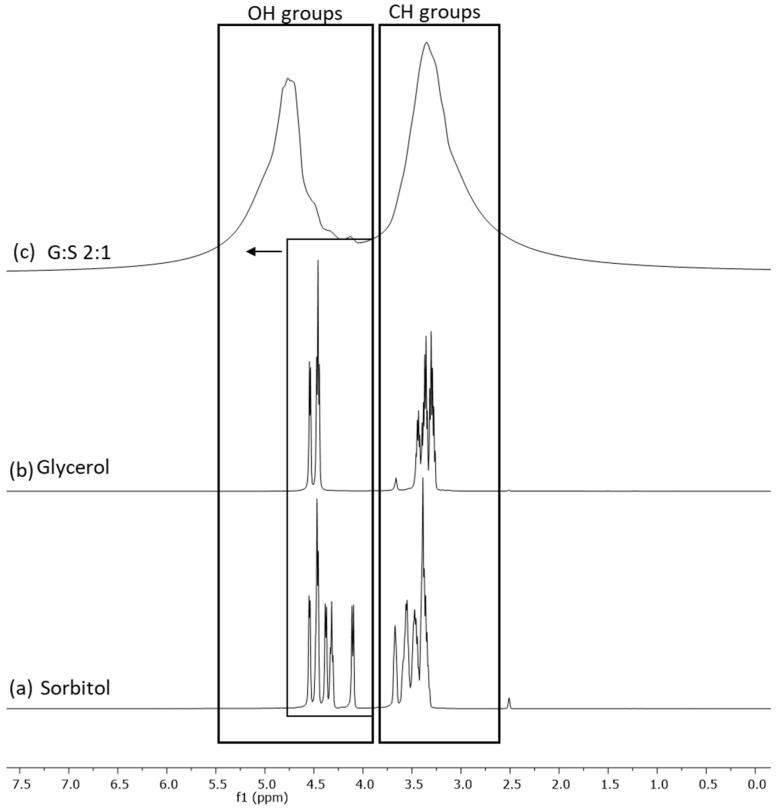
H^1^ NMR spectra of: (**a**) sorbitol, (**b**) glycerol, and (**c**) G:S 2:1 molar ratio.

NMR spectroscopy was performed for the identification of hydrogen-bonding interactions between the components of the LTTMs, the predominant type of interactions responsible for their formation. The NMR spectra of the C:A:W (Figure S2 in Supplementary material) were previously reported by the authors [29], where the results supported the presence of an altered hydrogen-bonding network in the ternary mixture. Regarding G:S and pure components, their H^1^ NMR spectra were here analysed. The proton peaks corresponding to the hydroxyl and alkyl groups of glycerol, sorbitol, and their mixture (G:S) were identified and are presented in Figure 2. Since the pure components have similar functional groups, their representative peaks are overlapped (Figure 2b,c). In the mixture (Figure 2a), this translates into two broad peaks comprehending the groups of the pure components. However, although the alkyl peak of the mixture is aligned with the ones from the pure components, the hydroxyl band of G:S shifted downfield in respect to the same groups of the individual compounds. This shift is an indicator of hydrogen interactions established between the hydroxyl groups of glycerol and sorbitol.

In addition to NMR, FTIR spectroscopy is also widely used for the identification of functional groups, whose signal may shift when molecular interactions are established.

**Figure 3:**
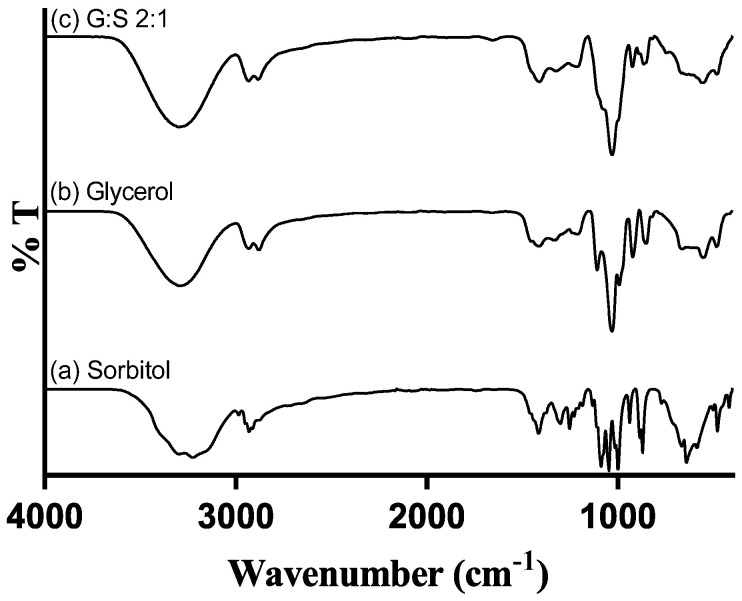
FTIR spectra of: (**a**) pure sorbitol; (**b**) pure glycerol; and (**c**) G:S 2:1 molar ratio.

The FTIR spectra of C:A:W (Figure S3 in supplementary material) were presented and discussed in a previous paper and confirmed the establishment of interactions between their components [42]. Regarding G:S, the spectra of the pure components and their mixture are presented in Figure 3. The bands between 3000 and 3500 cm^−1^ correspond to O-H stretching, while the C-H stretching is comprehended between 2800 and 3000 cm^−1^. Further, O-H bending vibrations can be identified at 1411–1415 cm^−1^ and C-OH stretching vibrations within 950 to 1150 cm^−1^. These spectra and respective assignments [43] are concordant to others previously reported for glycerol [41,44,45] and sorbitol [38,41,46,47]. Given that glycerol and sorbitol are composed by the same functional groups (hydroxyls and alkyls), their pure spectra have overlapping bands (Figure 3a,b). Thus, the G:S spectra (Figure 3c) have a combined profile of both molecules, predominantly glycerol since it is the main component. This confirmed that neither glycerol nor sorbitol suffered chemical changes upon mixing and heating. Furthermore, the hydrogen network change supported by NMR was not evidenced in the FTIR spectra since no band shifts were observed for the G:S systems in respect to the pure molecules. This probably occurred because the new hydrogen interactions between glycerol and sorbitol did not significantly alter the strength and dipolar moment of the pool of interactions, and, thus, their vibration frequency did not change.

### 3.4. Cytotoxicity

The cytotoxicity assay was performed in a L929 cell line, aiming to evaluate the biocompatibility of the G:S system in normal cells, according to the ISO 10993 [30]. The cytotoxicity of C:A:W and respective pure components was previously studied in a CACO-2 cell line [18]. Despite being different cell lines, it was expected that the IC_50_ determined (Table 1) would be in the same order of magnitude. As is possible to observe in Table 1, the pure glycerol and sorbitol presented a higher IC_50_ than citric acid and L-arginine. In terms of the respective mixtures, this translated into an IC_50_ for C:A:W about 25-fold lower than the one of G:S. Moreover, regarding the glycerol and sorbitol components and mixtures, the lowest IC_50_ was obtained for sorbitol, which may be attributed to osmotic stress induced in cells [48].

**Figure 4:**
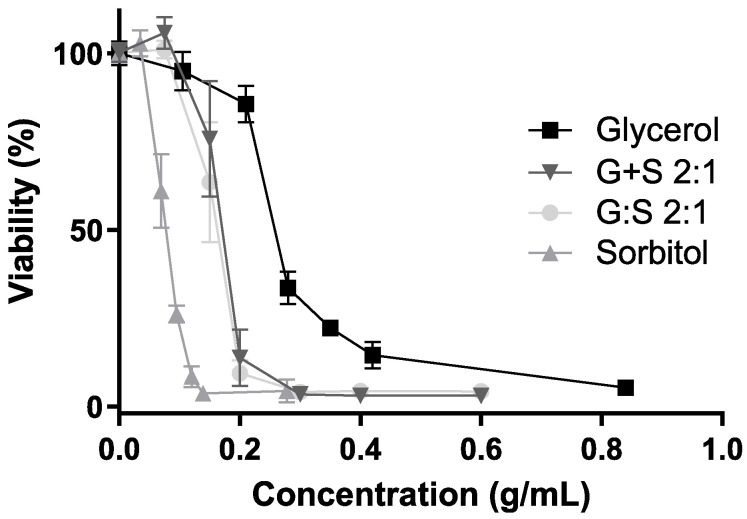
Cell viability (%) for different concentrations of glycerol (square), sorbitol (triangle), G:S 2:1 (circle), and G + S 2:1 (inverted triangle). No significant differences between the viability profiles of G:S and G + S (*p*-value > 0.8).

The cell viability results for several concentrations of G:S, the physical mixture G + S, and their pure components are further represented in Figure 4. Sorbitol showed maximum cell viability up to 0.034 g/mL, while glycerol presented a viability higher than 90% for concentrations up to 0.105 g/mL. The results of the pure components were concordant with others reported for a different cell line (human HEK-293), in which neither glycerol mor sorbitol were toxic up to 100 mM (i.e., 0.009 and 0.018 g/mL, respectively) [26]. Still, their cytotoxicity may vary depending on the cell type, and it is known to be time and dose dependent [48,49]. Therefore, the formulations of G:S can be adapted to the type of application required, for example, low doses for applications to healthy cells or high doses when targeting abnormal cells, like cancer.

Regarding G:S and the physical mixture G + S, maximum cell viability was observed up to a concentration of 0.075 g/mL (Figure 4).

Furthermore, it was reported that supramolecular complexes may have different results in regards to respective non-interacting pure components [13,25,26]. Hence, given the possibility of the systems’ G:S to have a supramolecular network, characteristic of some LTTM, like DES, it is important to compare the system with the physical mixture of the individual components at the same proportion (G + S). Since, upon dilution, both G:S and the respective physical mixture G + S have equivalent influence in the cell viability (Figure 4), it can be postulated that if G:S has a supramolecular complex organization, it is disrupted after aqueous dilution in the range of concentrations herein tested. Despite that some DES can incorporate water up to certain amounts, it is commonly reported that above a certain quantity, water dissolves the pure components, disrupting their supramolecular network [50,51,52], which might be the case and explain the observed results. Thus, despite that the G:S dilutions studied in the cytotoxicity assay were probably aqueous solutions, no further conclusions were possible to obtain in regard to the network arrangement of higher concentrations or non-diluted forms of G:S.

In sum, from the results herein acquired, the G:S system can be considered biocompatible upon certain amounts and depending on the type of administration and pharmacokinetics.

### 3.5. Viscosity

**Figure 5:**
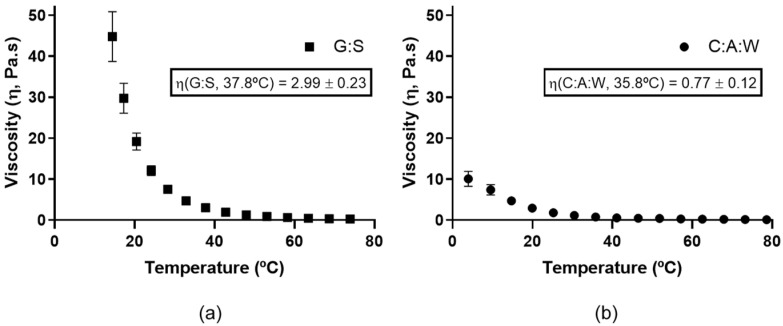
Dynamic viscosity for: (**a**) G:S from 80 to 4 °C; (**b**) C:A:W from 4 to 80 °C.

The viscosity is a parameter known to highly influence the dissolution rate of a solute in a solvent. Therefore, the characterization of this parameter in new liquid systems is highly important for their choice as a delivery vehicle and suitable application. The dynamic viscosity of C:A:W and G:S was studied as a function of temperature, and the results are represented in Figure 5. While handling the G:S system, it was observed that its viscosity depends on time and temperature. This suggests a reversible physical rearrangement of the structure, which is consistent with the observation of its gelification over time at low temperatures (<40 °C). However, at 80 °C the influence of these factors was no longer observed, guaranteeing consistent initial properties. Thus, the viscosity profile of G:S was performed from 80 to 4 °C to ensure reproducibility. On the other hand, for the C:A:W it was performed from 4 to 80 °C, to avoid the influence of water evaporation.

As is possible to observe, the viscosity decreases as the temperature increases for both systems. Moreover, it can be observed that the G:S (Figure 5a) has a higher viscosity than C:A:W (Figure 5b). At values close to the physiologic temperature, G:S presented a viscosity value around 2.99 Pa.s (37.8 °C), which is 3.9 times higher than the one of C:A:W (0.77 Pa.s, 35.8 °C). Since the dissolution rate is slower for higher viscosities [53], this translates into diffusion rates slower for G:S in comparison to C:A:W. Therefore, the use of C:A:W as a delivery system would probably be more adequate for applications that require rapid dissolution rates, while G:S may be more advantageous for sustained release applications.

### 3.6. Solubility Insight

**Figure 6:**
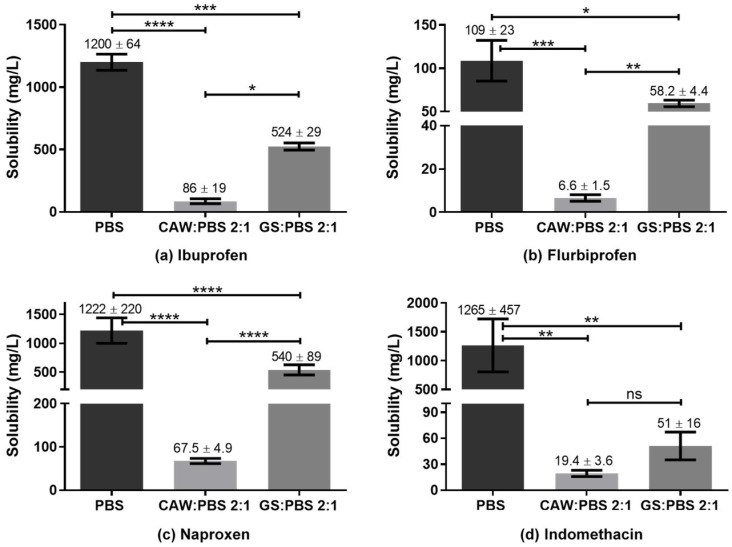

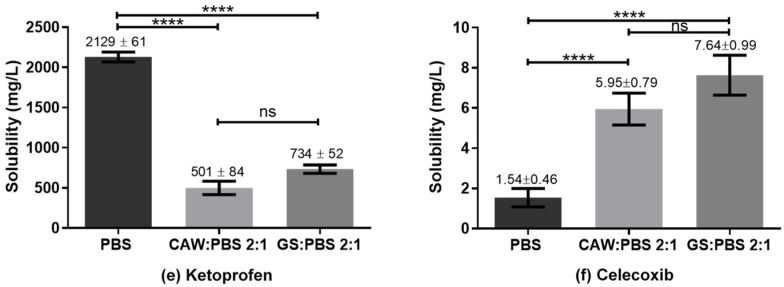
Solubility of NSAIDs in PBS, CAW:PBS 2:1, and GS:PBS 2:1 (*w*/*w*). **** *p* < 0.0001; *** *p* ≤ 0.0007; ** *p* ≤ 0.01; * *p* ≤ 0.03; ^ns^ *p* > 0.1.

The goal of the present study was to evaluate if G:S and C:A:W could be used as therapeutic liquid mixtures by carrying different NSAIDs and improving their solubility in respect to PBS. For this, the solubility of the drugs was determined in concentrated G:S and C:A:W media, with a ratio of 2:1 (*w*/*w*) GS:PBS and CAW:PBS. This ratio was set to mimic formulations for localized administration (e.g., intra-articular injection) and to screen whether the G:S and C:A:W systems have potential to increase the NSAIDs’ solubility in physiological media. The results obtained for the six NSAIDs tested are summarized in Figure 6.

Reported solubility values for the different NSAIDs vary in some cases to a great extent. Nonetheless, according to our experiments, the solubility obtained for ibuprofen in PBS was lower than those reported in the literature (2000 to 7300 mg/L) [54,55,56,57]. For indomethacin the value obtained was concordant with Ivanova et al. (1150–3170 mg/L) [58] but much higher than that indicated by Cayman (50 mg/L) [59]. Ketoprofen’s solubility in PBS was within the range reported by Cayman (500 mg/L) [60] and Shohin et al. (3310 mg/L) [61] but lower than the one of Felton et al. [56]. For flurbiprofen, the determined PBS solubility was in accordance with Sadik et al. (130 ± 40 mg/L) [62] and Daravath et al. (98 ± 10 mg/L) [63], despite the heterogeneity of values reported (98 to 4445 mg/L) [56,62,63,64,65]. The value obtained for naproxen was in agreement with Cayman (1000 mg/L) and Kumar et al. (750 ± 250) mg/L, being lower than Felton and co-authors’ value (4259 mg/L). In turn, celecoxib presented a PBS solubility equivalent to Abu-Diakes et al. (1.58 ± 0.03 mg/L) but lower than Rawat and co-workers (47.15 mg/L).

While the experimental values of the NSAIDs’ solubility in PBS herein determined were in accordance with part of the literature, there were also visible discrepancies within the values reported. These differences might be due to several factors such as drugs’ particle size, polymorphism, purity, precision of the quantification method, time and temperature of the assay and storage, and phosphate concentration of the buffer, among others.

Regarding the comparison of drugs’ solubility in the studied media, all NSAIDs presented a higher solubility in PBS than in the 2:1 GS:PBS or CAW:PBS, except for celecoxib. For those NSAIDs, the C:A:W seemed to strongly act as anti-solvent. On the contrary, the CAW:PBS 2:1 and particularly the GS:PBS 2:1 increased the celecoxib solubility by 4-fold and 5-fold, respectively. To better understand these observations, polarity and pH measurements were carried out.

**Figure 7:**
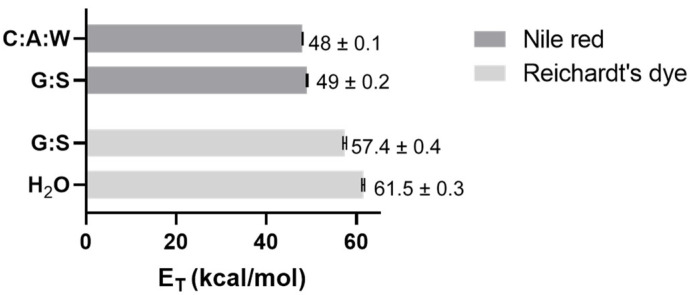
E_T_ values obtained for C:A:W, G:S, and H_2_O using Nile red and/or Reichardt’s dye.

The polarity is one of the parameters considered when evaluating the capacity of a solvent to dissolve a solute, through the principle ‘like dissolves like’. Thus, the polarity of G:S and C:A:W was estimated and compared to water as standard solvent and equivalent of PBS. This was carried out using two solvatochromic probes whose absorption spectra shift depending on the polarity of the solvent. The Nile red dye was used as it is more appropriate for apolar solvents while the Reichardt’s dye is better for polar solvents. According to the energy transition values (E_T_) calculated from Equation (2), it was possible to establish the relative polarity of the studied systems. Since water is extremely polar, it was only analysed by the Reichardt’s dye. Moreover, for the C:A:W, only the Nile red was suitable because the Reichardt’s dye reacts with the carboxylic acid groups from citric acid and L-arginine [66,67]. The results obtained are summarized in Figure 7.

From the Nile red analysis it can be stated that C:A:W is slightly more polar than G:S, whereas the Reichardt’s dye data indicate that H_2_O is more polar than G:S. Therefore, their relative polarity can be ordered from the highest to the lowest as follows: H_2_O > C:A:W > G:S. Upon dilution in physiological media, their relative polarity will vary accordingly.

The pH is also an important parameter when evaluating the solubility of certain compounds because it influences their structure according to the pKa of the functional groups, which can dictate a higher or lower solubility. Therefore, the pH of PBS and the several ratios between GS:PBS and CAW:PBS tested in the solubility assay are summarized in Table 2. As observed, both PBS and GS:PBS mixtures present a neutral pH while the CAW:PBS systems have an acidic pH.

**Figure 8:**
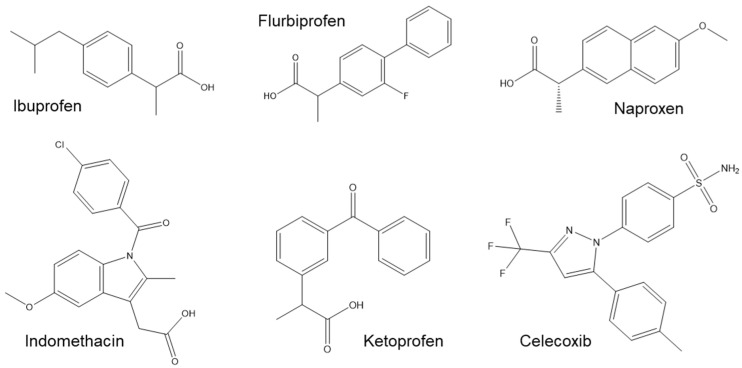
Chemical structure of ibuprofen, flurbiprofen, naproxen, indomethacin, ketoprofen, and celecoxib.

In terms of polarity, celecoxib had higher solubility in the less polar solvent (GS:PBS) while the other NSAIDs presented the highest solubility in the more polar solvent. This might be explained by their differences in terms of chemical composition (Figure 8).

Although celecoxib has some polar groups such as the fluorines, it has also three non-polar rings and, thus, the final polarity balance might be closer to the one of GS:PBS. In turn, a common feature between ibuprofen, indomethacin, ketoprofen, flurbiprofen, and naproxen is their carboxylic acid group, which is not present in celecoxib. This functional group contributes to their higher polarity and clarifies the deep decrease in solubility observed for the CAW:PBS media, which are related to its pH. The pKa of the NSAIDs’ carboxylic acids varies within 3.9 to 5.3 [68,69,70]. Therefore, at the acidic pH of CAW:PBS media (3.5), the COOH is mostly protonated, decreasing the solubility of those drugs in the mixture. On the contrary, at the neutral pH of PBS and GS:PBS, the COOH group is deprotonated, increasing the drugs’ solubility in those media. Another interesting feature for these group of drugs is that, while all of them present a mean solubility higher for GS:PBS than CAW:PBS, the difference was not statistically significant for indomethacin and ketoprofen. This was probably caused by their additional polar groups, mainly the carbonyl (C=O), which increased their polarity, decreasing the solubility in GS:PBS.

Regarding celecoxib, within the studied parameters, its solubility was mostly dependent on polarity since its pKa is about 11 [71] and, thus, it was in the non-charged form at all pHs.

Overall, celecoxib was the only NSAID that presented an increased solubility of about 4-fold for the CAW:PBS 2:1 and 5-fold for the GS:PBS 2:1, in comparison to PBS alone. This confirmed that concentrated formulations of C:A:W and G:S may be suitable for localized administration of celecoxib with improved efficiency.

**Figure 9:**
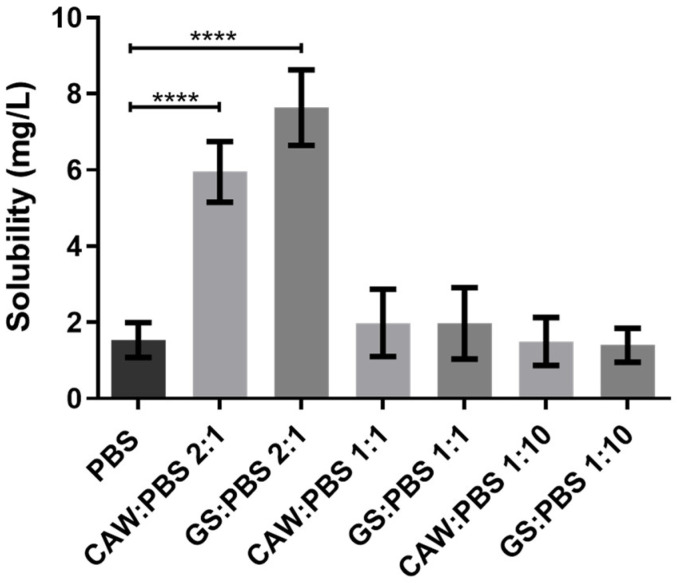
Solubility of celecoxib in PBS and different ratios of GS:PBS and CAW:PBS (2:1, 1:1, and 1:10, *w*/*w*). Statistical differences between PBS, CAW:PBS 2:1, and GS:PBS 2:1 (**** *p* < 0.0001). The differences between CAW:PBS 1:1, GS:PBS 1:1, CAW:PBS 1:10, GS:PBS 1:10, and PBS were not significant (*p* > 0.9999).

Since it presented an improved solubility in those media, celecoxib was further tested in 1:1 and 1:10 (*w*/*w*) ratios of GS:PBS and CAW:PBS to mimic applications where the formulations are diluted in the physiological media (e.g., oral or systemic). As observed in Figure 9, there were no statistically significant differences between the solubility determined in PBS, CAW:PBS 1:1, GS:PBS 1:1, CAW:PBS 1:10, and GS:PBS 1:10. Although the 2:1 (*w*/*w*) formulations of CAW:PBS and GS:PBS increased celecoxib’s solubility, upon dilution in PBS, the dissolution advantage conferred by C:A:W and G:S was lost. Therefore, these systems are probably not suitable for administration pathways that are diluted in the physiological media as this would possibly cause the drugs to precipitate.

Considering the global solubility results, it can be stated that, from all the therapeutic liquid mixtures studied, the G:S or C:A:W with celecoxib are the ones with prospects for further development towards localized administration.

## 4. Conclusions

In this work, G:S and C:A:W mixtures were explored in terms of their properties and ability to improve NSAIDs’ solubility in therapeutical applications. To the best of our knowledge, the G:S LTTM and respective characterization were reported for the first time. Within the NSAIDs studied, GS:PBS and CAW:PBS 2:1 (*w*/*w*) formulations improved the solubility of celecoxib 4-fold and 5-fold in comparison to PBS, respectively. In general, this is a promising achievement for local applications, with concentrated therapeutic liquid systems.

In sum, the developments herein achieved represent a starting point for the optimized production of a therapeutic formulation with increased celecoxib solubility, which translates into higher efficiency and reduced adverse effects. For the pharmaceutical industry it also represents a valorisation of the commercial drug. Moreover, the compounds used in the formulations are commonly used as excipients and, thus, could avoid the time associated with the regulatory entities to reach the market.

## Figures and Tables

**Table 1:** IC_50_ determined for glycerol, sorbitol, G:S 2:1, and G + S 2:1 (molar ratio), represented in mean ± SD. * The IC_50_ values for citric acid, L-arginine, and C:A:W 1:1:7 were determined elsewhere [18] in a CACO-2 cell line.

Sample	IC_50_ (g/mL)
Glycerol	0.2583 ± 0.0225
Sorbitol	0.0751 ± 0.0064
G:S 2:1	0.1564 ± 0.0113
G + S 2:1	0.1656 ± 0.0154
Citric acid	0.0007 *
L-arginine	0.0177 *
C:A:W 1:1:7	0.0062 *

**Table 2:** The pH values for PBS and mixtures of CAW:PBS and GS:PBS at different ratios (*w*/*w*).

Media	pH
PBS	7.4
CAW:PBS 2:1	3.5
GS:PBS 2:1	7.1
CAW:PBS 1:1	3.5
GS:PBS 1:1	7.2
CAW:PBS 1:10	3.6
GS:PBS 1:10	7.3

## Supplementary Materials

The following are available online at www.mdpi.com/article/10.3390/pharmaceutics13101620/s1. Figure S1: DSC thermogram of C:A:W 1:1:7 (molar ratio). Figure S2: H^1^ NMR spectra of (a) citric acid:H_2_O pH = 3.5; (b) citric acid:H_2_O 1:7; (c) citric acid:L-arginine:H_2_O 1:1:7.; (d) L-arginine:H_2_O pH = 3.5. Figure S3: FTIR spectra of (a) pure L-arginine (orange line); (b) L-arginine:H_2_O 1:7 mol (light blue line); (c) L-arginine:H_2_O pH = 3.5 (green line); (d) citric acid:L-arginine: H_2_O 1:1:7 mol (red line); (e) citric acid: H_2_O pH 3.5 (brown line); (f) pure citric acid (dark blue line).
