# Supplementary material for: Therapeutic Liquid Formulations Based on Low Transition Temperature Mixtures for the Incorporation of Anti-Inflammatory Drugs
Source: Pharmaceutics. 2021 Oct 5;13(10):1620. doi: 10.3390/pharmaceutics13101620 (PMC8540160; doi:10.3390/pharmaceutics13101620)
Supplement: Supplementary file 1 [file pharmaceutics-13-01620-s001.zip › pharmaceutics-1359238-supplementary.pdf]

pharmaceutics

MDPI

Supplementary Materials: Therapeutic Liquid Formulations Based on Low Transition Temperature Mixtures for the Incorporation of Anti-Inflammatory Drugs

Ana Roda and Alexandre Paiva Nand Ana Rita C. Duarte *

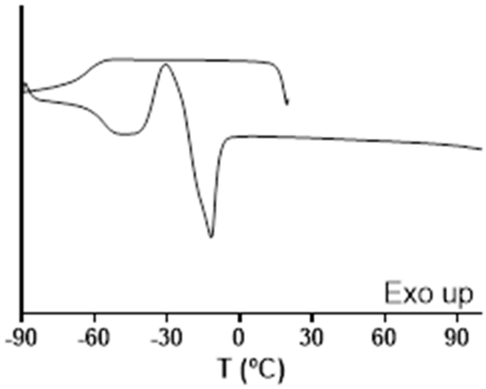

Figure S1. DSC thermogram of C:A:W 1:1:7 (molar ratio). Data from Roda et al. (ref. [29] in main article).

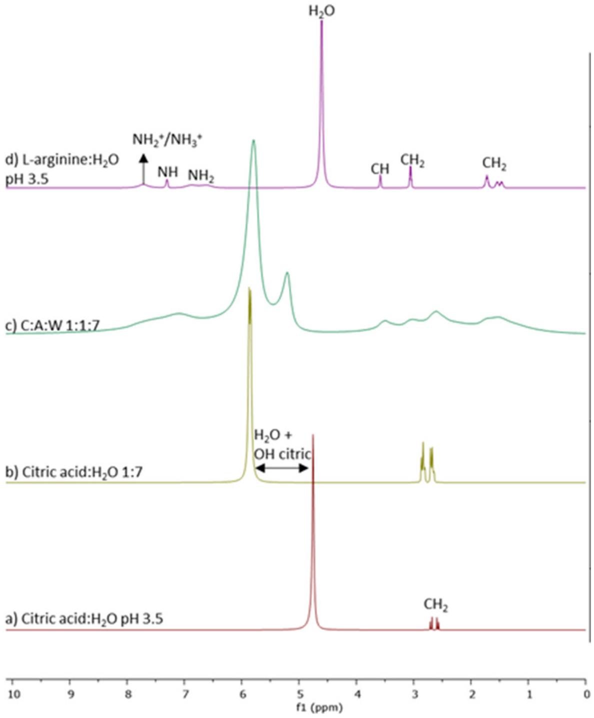

Figure S2. "H1 NMR spectra of a) citric acid:H2O pH=3.5; b) citric acid:H2O 1:7; c) citric acid:L-arginine:H2O 1:1:7"; d) L-arginine:H2O pH=3.5. Figure adapted from data published in Roda et al. (ref. [29] in main article); consult the cited manuscript for detailed discussion.

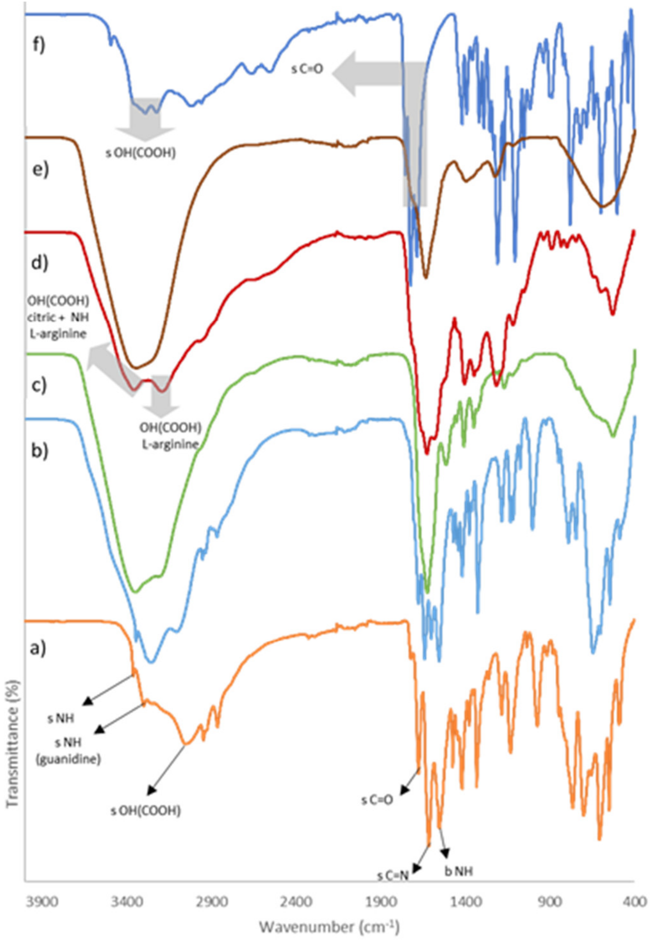

Figure S3. "FTIR spectra of a) pure L-arginine (orange line); b) L-arginine:H2O 1:7 mol (light blue line); c) L-arginine:H2O pH=3.5 (green line); d) citric acid:L-arginine: H2O 1:1:7 mol (red line);" e) citric acid: H2O pH 3.5 (brown line); "f) pure citric acid (dark blue line). "s" and "b" refer to stretching and bending vibrations, respectively." Figure adapted from data published in Roda et al. (ref. [29] in main article). Consult the cited manuscript for detailed discussion.
